# Ovarian Hormones and Addictive Behaviour Vulnerability: Insights From Preclinical Studies

**DOI:** 10.1111/adb.70046

**Published:** 2025-06-08

**Authors:** Leonardo Vázquez‐Morales, Gisela Aguirre, Tania Molina‐Jiménez, Rossana C. Zepeda, Óscar López‐Franco, Mónica Flores‐Muñoz, Claudia Juárez‐Portilla

**Affiliations:** ^1^ Facultad de Biología Universidad Veracruzana Xalapa Veracruz Mexico; ^2^ Laboratorio de Neurobiología de la Conducta y Procesos Neuroquímicos, Centro de Investigaciones Biomédicas Universidad Veracruzana Xalapa Veracruz Mexico; ^3^ Facultad de Química Farmacéutica Biológica Universidad Veracruzana Xalapa Veracruz Mexico; ^4^ Laboratorio de Biomedicina Integral y Salud, Centro de Investigaciones Biomédicas Universidad Veracruzana Xalapa Veracruz Mexico; ^5^ Laboratorio de Medicina Traslacional, Instituto de Ciencias de la Salud Universidad Veracruzana Xalapa Veracruz Mexico

**Keywords:** addiction, drug consumption, oestrogen, preclinical studies, progesterone, psychoactive substances, substance use disorder, systematic review

## Abstract

Substance use disorder constitutes a global health challenge. Preclinical investigations into addiction heavily rely on animal models to explore the underlying biological mechanisms of addictive disorders, with a particular emphasis on understanding the etiological factors influencing drug intake. Exploring sex differences across various phases of addiction has revealed a heightened vulnerability in females. This study systematically reviews the impact of ovarian hormones on the consumption of psychoactive substances in rodents, adhering to the PRISMA 2009 protocol. Our findings underscore the significant role of ovarian hormones, particularly oestrogen, in augmenting drug consumption among female rodents. Notably, with heroin, it was observed that progesterone, rather than oestrogen, facilitated increased consumption in female rodents. The susceptibility to addiction influenced by oestrogen is accentuated across distinct phases, and the molecular mechanisms form a complex interplay that significantly influences addictive behaviours. By bringing together these findings, we aim to establish a strong foundation for future studies. This work may guide clinical investigations in developing more effective prevention or treatment strategies that address the unique vulnerabilities of females to substance use disorders.

## 1 Introduction

Pathological abuse of psychoactive substances (PASs) constitutes a mental disorder leading to both short‐ and long‐term alterations in the central nervous system (CNS) and overall physiological functioning. PAS addictive behaviour presents a global health challenge with profound societal, political and economic ramifications [1, 2]. The study of addictive behaviour has been instrumental in identifying risk factors contributing to the onset of this mental illness and formulating effective prevention strategies [3]. Preclinical investigations have delineated the specific brain regions implicated in the progression of drug addiction, revealing neuroadaptive changes following chronic substance use. These changes result in diminished sensitivity, fostering the development of maladaptive patterns, such as compulsive PAS consumption despite adverse effects. This behaviour ultimately harms users' health and can lead to fatal outcomes [4, 5].

Seminal work by Roberts et al. in the 1980s, followed by studies from Lynch et al. in the late 1990s and early 2000s, highlighted the critical role of reproductive cycles and hormonal fluctuations in modulating PAS consumption, reinforcing motivation and driving drug‐seeking behaviour in female rodents [6, 7, 8, 9, 10, 11, 12]. Advancements in experimental models within preclinical studies have unveiled phenotypic individualities or ‘risk groups’ associated with substance use disorder, including traits such as high compulsivity, impulsivity and variations related to age and sex [13, 14]. Notably, sexual differences have been highlighted, with females exhibiting greater sensitivity and lower aversion to drugs [15]. This phenomenon is directly linked to the influence of ovarian hormones, particularly in the acute stimulation induced by PAS and certain opioids. Arunogiri et al. reported that these hormonal influences are especially prominent during key phases of the addictive cycle, such as acquisition, escalation and relapse. Examining hormonal effects on specific addiction phases is crucial for assessing susceptibility to substance use disorders, understanding underlying mechanisms and developing targeted treatments [16].

Considering this, our primary objective was to conduct a systematic review of the most recent findings on the correlation between ovarian hormones, specifically progesterone (PRO) and estradiol (E2), and the consumption of PAS during distinct phases of the addictive cycle in murine models.

## 2 Methods

**FIGURE 1:**
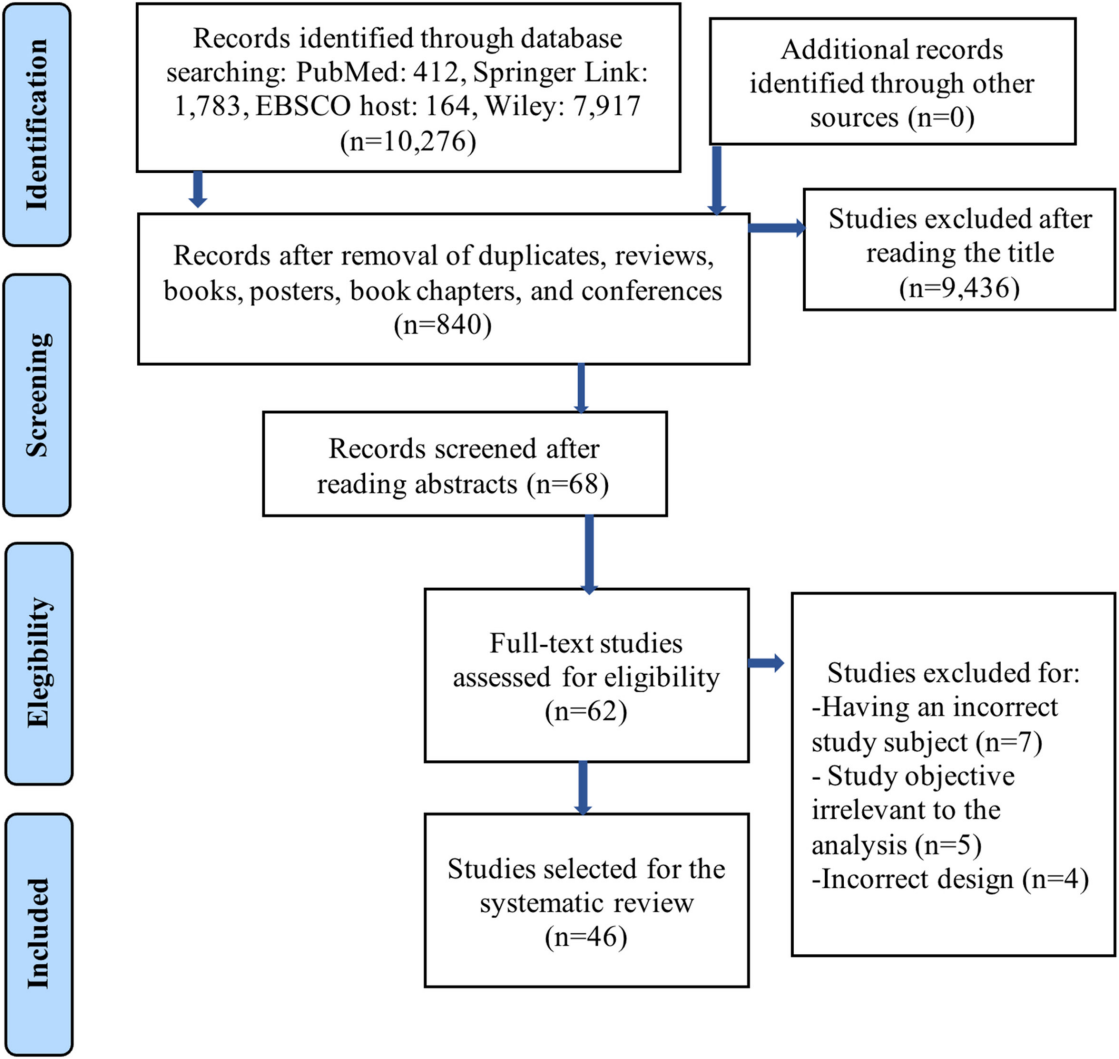
PRISMA flow diagram. Flowchart of the studies included after passing the screening and selection process.

This systematic review adheres to the PRISMA guidelines for reporting systematic reviews and meta‐analyses [17], ensuring a comprehensive and reproducible documentation of the research process (see Figure 1). The PRISMA guidelines were selected to uphold the transparency and quality of the study, providing a structured framework for electronic searches and overall reporting.

### 2.1 Eligibility

#### 2.1.1 Inclusion Criteria

Searching was conducted three times: February, December 2023 and March 2024. This review considered original experimental studies published within the last 16 years until March 2024. Articles written in English or Spanish, providing full‐text descriptions of preclinical murine models used in experimental studies investigating the influence of ovarian hormones on PAS intake across various addiction stages, were included.

#### 2.1.2 Exclusion Criteria

Documents such as reviews, books, book chapters, symposiums and articles that did not describe or exemplify any phase of addictive behaviour were excluded. Studies employing animal models other than rodents were also discarded. In unclear or unreported data cases, attempts were made to contact the authors for clarification.

#### 2.1.3 Reference Sources, Keywords and Data Extraction

Literature searches were conducted in EBSCO, PubMed, Springer Link and Wiley databases. Following the method outlined [16], three main concepts were established, each comprising different keywords: (1) ‘Females’ (participants of the study) AND (2) ‘Ovarian hormones’ OR ‘sex differences’ OR ‘estrogen’ OR ‘progesterone’ (modulator) AND (3) ‘Psychoactive substance’ OR ‘Relapse’ OR ‘Craving’ OR ‘Abstinence’ OR ‘Addiction’ (related to addictive behaviour). The filter ‘preclinical models’ was also applied in PubMed, while ‘Animal models’ was used in the other platforms as the fourth concept to avoid clinical studies.

Two authors independently conducted searches, reviewing titles and abstracts on the web. Articles needing to meet the inclusion criteria were removed at this stage. Subsequently, two additional authors selected the final studies based on eligibility criteria after reviewing the full texts.

#### 2.1.4 Data Extraction

The information extraction for subsequent analysis followed the methodologies outlined by Arunogiri et al. [16] with modifications based on Lynch et al. [18]. Data was then subdivided into observational and interventional studies. Observational studies included those examining one or more phases of addictive behaviour and their correlation with ovarian hormones in intact animals cycling freely without hormonal treatments. Interventional studies had at least one group of gonadectomised animals with or without exogenous hormone treatment: PRO, oestrogen (E2) or both. The phase of the addictive cycle to be modelled in each study was also considered, followed by the type of comparison (i.e., sex difference analysis or hormonal type evaluated). Supported by Lynch et al. with a modification, it also included species and strain used, PAS, administration paradigm, a summary of key results, the country where the research laboratory was located and the source (year of publication and authors) [18]. The standardization of experimental subjects' age across the 46 studies followed the methodology outlined by Ghasemi et al. [19] with modifications based on McCutcheon and Marinelli [20] and Radulescu et al. [21]. Studies that reported animal age in days were directly classified using the criteria established by Ghasemi et al. [19]. When only body weight was provided, the conversion was performed using McCutcheon and Marinelli [20] in conjunction with Ghasemi et al. [19]. For studies using mice, age classification was determined following Radulescu et al. Additional variables included species, strain and age range reported. A supporting information table (see Table S1) summarizing this information has been included to enhance transparency and consistency across studies. Additionally, a supplementary analysis was conducted with the extraction of neurochemical mechanisms proposed by the authors for ovarian hormone modulation, as described in the key outcomes.

#### 2.1.5 Methodological Quality and Risk of Bias Assessment

The risk of bias was evaluated using a 13‐point instrument modified from Hooijmans et al., OHAT and Recommendations for Ensuring Good Scientific Inquiry tools [22, 23, 24]. The original version of SYRCLE's RoB (Systematic Review Centre for Laboratory Animal Experimentation's risk of bias) by Hooijmans et al. was employed for assessing bias in various methodological domains of the obtained preclinical records, covering selection, comparison and results [22]. Additionally, the RoB tool modified by OHAT and the Recommendations for Ensuring Good Scientific Inquiry tool were used to assess the quality of the methodological strategies and results in the studies [23, 24]. Two independent reviewers participated in the methodology quality and risk of bias assessment to enhance efficiency. Condensed scores from double‐blind and lead‐author responses were reported.

## 3 Results

### 3.1 Study Selection

Utilizing the methodology mentioned above, 10 276 articles were identified and distributed across databases: 412 from PubMed, 1783 from Springer Link, 164 from EBSCO and 7917 from Wiley. After excluding reviews, books, posters, book chapters and conference materials, 840 records remained. After evaluating titles, duplicate studies and those deemed unsuitable due to divergent topics, alternative biological models or the absence of addiction modelling were removed. This led to the inclusion of 46 studies for the systematic review (Figure 1). No additional searches were employed to integrate studies into the review.

### 3.2 Study Characteristics

Of the 46 studies obtained, 28 were observational (including 26 purely observational and two with at least one interventional experiment), while 18 were exclusively interventional (excluding the two counted as observational). Table 1 summarizes the findings from observational studies, whereas Table 2 presents the findings from interventional research.

**TABLE 1:** Observational studies.

Addiction phase	Comparison	PAS	Administration model	Outcome	Reference
Acquisition	Sex and hormonal differences	EtOH	SA	Alcohol consumption: Diestrus F > M; estrus F = M	[25]
	Sex differences	COC	SA	Longer binge: F > M	[26]
	Sex and hormonal (age‐related) differences	COC, KET, MDMA and THC	SA	Behavioural sensitization by sex: F + KET > M + KET; F + THC = M + THC	[27]
				Behavioural sensitization by age: FAdult + COC = FAdolest + COC; FAdult + KET = FAdoles + KET; FAdoles + MDMA > FAdult + MDMA; FAdoles + THC = FAdult + THC	
	Sex and administration model differences	OXY	CPP and FA	Changes in the opioid system: CPP > FA; F > M	[28]
	Sex differences	MA	FA	Acute locomotor response: F > M	[29]
	Sex differences	MDMA	FA (MDMA); SA (ICSS)	Behavioural and neurochemical sensitivity to MDMA and ICSS: F > M	[30]
	Sex and model of administration differences	HER	SA LgA vs. SA IntA	Intake; IntA > LgA; F = M	[31]^a^
	Sex differences	HER	SA	Drug acquisition: F > M	[32]^a^
	Sex differences	OXY	SA	Drug acquisition: F > M	[33]^b^
Maintenance	Sex differences	COC	SA	Drug‐seeking: Diestrus F = M Estrus F > M	[34]^b^
	Sex and hormonal differences	COC	CPP	Reward: Estrus > diestrus; F > M	[35]
	Sex differences	NIC	SA (PR)	NIC consumption induced by YOH: F > M	[36]
	Sex differences	EtOH	SA + stress model	Drinking under stress induced by wheel access blockage: F > M	[37]
	Sex and strain differences	Hallucinogens 1‐(2,5‐dimethoxy‐4‐iodophenyl)‐2‐aminopropane (DOI) hydrochloride and (R)‐(+)‐α‐(2,3‐dimethox‐yphenyl)‐1‐[2‐(4‐fluorophenyl)ethyl]‐4‐piperinemethanol	FA	Sensitivity to hallucinogens per strain: C57BL6J: F > M	[38]
				129S6/SvEv: F = M	
Motivation	Sex and hormonal differences	COC	SA + runway model	Motivation: F > M	[39]
				Aversion: No‐estrus > estrus	
	Sex differences	OXY	CPP	Greater OXY‐seeking CPP: F > M	[40]
	Sex and age differences	MA	CPP and CPA	CPP: Adoles > adults; F = M	[41]
				CPA: Adults > adoles; M > F	
	Sex and administration model differences	4MMC	CPP	Conditioning to classic CPP: F > M	[42]
			CPP; CPP + SC	CPP + SC > CPP	
			CPP; CPP + CMUS	CPP + CMUS > CPP	
	Sex differences	EtOH	CPP‐CIE + footshock	CPP‐CIE: F > M	[43]
				Aversion resistance: F > M	
	Sex differences	THC	FA + behavioural test battery	Reward: F > M	[44]
				Behavioural despair: F > M	
				Anhedonia: F = M	
	Sex differences	COC	SA (LgA vs. IntA)	Consumption under LgA: F > M	[45]
				Consumption under: IntA: F = M	
Escalation	Sex differences	COC	SA (PR)	Motivation under reinforcement schedule: F > M	[46]^a^
	Sex and hormonal differences	COC	SA (PR)	Greater drug breakpoints: Estrus F > proestrus F; M: stable	[47]
		HER	SA	Drug intake: F > M	
				Drug intake: No‐estrus F > proestrus, estrus F	
	Sex and administration model differences	MA	SA (LgA vs. ShA)	Escalated intake: F > M under LgA.	[48]^a^
	Sex differences	MA	SA	Increased drug intake: M > F	[49]^a^
Extinction	Sex differences	MA	SA	Memory deficits following extinction: F = M.	[48]^a^
	Sex differences	COC	SA	Motivation during withdrawal: F > M	[46]^a^
	Sex, hormonal and model of administration differences	HER	SA (LgA vs. IntA)	Greater cue‐induced craving: InA > LgA	[31]^a^
				HER seeking: F > M, estrus cycle independent	
	Sex differences	HER	SA + BU08028	Reduction of drug‐seeking under BU08028 treatment: F = M	[50]^a^
	Sex differences	MA	SA	Drug‐seeking: M = F	[49]^a^
	Sex and age differences	MORPH	FA	Duration of withdrawal on aged rats F = M and adult rats M > F	[51]
	Sex differences	EtOH	SA + alternate punishment and nondrug reinforcement	Greater suppression due to punishment: M > F	[52]^a^
Reinstatement	Sex differences	MA	SA (low and high doses)	Reinstatement: Low > high; F > M	[48]^a^
	Sex differences	COC	SA + histamine	Reinstatement resistant to punishment with histamine: F > M	[46]^a^
	Sex differences	HER	SA + BU08028	Reinstatement under treatment: BU08028: F > M	[50]^a^
	Sex differences	HER	SA	Primed reinstatement: F > M	[32]^a^
				Cued reinstatement: F > M	
	Sex differences	OXY	SA	Cued reinstatement: F = M	[33]^b^
	Sex differences	EtOH	SA	Primed reinstatement: F > M	[52]^a^

Abbreviations: 4MMC: 4‐methylephedrone, CIE: chronic intermittent ethanol, CMUS: chronic mild unpredictable stress, COC: cocaine, CPP: conditioned place preference, EtOH: ethanol, F: female, FA: Forced abstinence, HER: heroin, ICSS: intracranial self‐stimulation, IntA: intermittent access, KET: ketamine, LgA: continuous access, M: male, MA: methamphetamine, MDMA: 4‐methylenedioxymethamphetamine, MORPH: morphine, OXY: oxycodone, PR: progressive‐ratio, SA: self‐administration, SC: social‐conditioning, THC: tetrahydrocannabinol.

^a^ More than one phase studied.

^b^ More than one phase studied, including interventional experiments described in Table 2.

**TABLE 2:** Interventional studies.

Addiction phase	Comparison	PAS	Administration model	Treatment	Outcome	Reference
Acquisition	Hormonal differences	COC	FA + AM251	E and AM251	Rewarding effect mediated by the endocannabinoid system in females: OVX + E > OVX + E + AM251	[53]
	Hormonal differences	NIC	CPP	TAM	CPP to the drug: F + TAM > F	[54]
	Sex and hormonal differences	CP55940	FA	E	Rewarding properties: OVX + E > F; OVX + E > M	[55]
	Hormonal differences	HER	SA	MIF and RAL	Drug intake in proestrus F: RAL > MIF	[56]
				E and PRO	Reduction in drug intake: OVX + E > OVX + PRO	
	Hormonal differences	NIC	SA	E1 and E2	Drug consumption: F > OVX; F > OVX + E1; F > OVX + E1 + E2	[57]
	Hormonal differences	OXY	SA	N/T	Drug acquisition: OVX > F‐sham; M‐sham > OCX	[33]^b^
Maintenance	Sex and hormonal differences	WIN	SA	N/T	Drug‐seeking: F > OVX > M	[58]^a^
	Strain differences				Drug‐seeking between strains: LE = LH	
	Hormonal differences	THC	SA	N/T	Behavioural and pharmacodynamic sensitivity: F > OVX	[59]
Motivation	Sex and hormonal differences	COC	SA	E	Preference of COC over food: F > M; F > OVX + ES > OVX	[39]
	Hormonal differences	MA	SA	E	Rapid behavioural sensitization to MA: OVX + ES > OVX	[60]
	Sex, hormonal and age differences	EtOH	CPP and CPA (high dose induced aversion)	N/T	CPP by age and sex: FAdult > AdultOVX; FAdoles > MAdoles	[61]
					CPP between estrus cycle phases: Proestrus > no‐proestrus	
					CPA: Fadult = AdultOVX = MAdul	
	Hormonal differences	FEN	SA and extended IntA	E	Greater consumption OVX + E > OVX	[62]^a^
Escalation	Sex and hormonal differences	EtOH	SA	E	Greater consumption: OVX > M	[63]
					Greater consumption under hormonal treatment: OVX + E > OVX	
	Sex and hormonal differences	COC + REMIFEN	SA	N/T	Greater demand of COC prior to REMIFEN consumption: Estrus > meta/diestrus; F = M	[64]
Extinction	Sex differences	COC	SA	E	Enhanced response: OVX + ES > OCX + ES	[65]
	Hormonal differences	HER	SA	E and PRO	Drug‐seeking under food restriction: F + PRO > F + ES	[66]
	Sex differences	COC	SA	N/T	Maintenance: F > M Extinction: F = M	[67]^a^
	Hormonal differences	FEN	SA	E	Greater weight loss: OVX + E > OVX	[62]^a^
Reinstatement	Sex and hormonal treatment differences	COC	SA + YOH vs. SA + YOH + ALLO	YOH, ALLO	Reinstatement alone: F > M	[34]^b^
					Reinstatement under treatment: F + YOH > F + YOH + ALLO; M + YOH = M + YOH + ALLO	
	Sex differences	EtOH	SA	YOH	Greater consumption: F + YOH > M + YOH	[68]
	Sex, hormonal and attenuation treatment differences	COC	SA	YOH	Greater reinstatement: F + YOH + cue> M + YOH + cue	[69]
					Reinstatement under stress‐activating drug alone: F + YOH = M + YOH	
					Reinstatement per estrus phase: Proestrus + YOH + cue > no‐proestrus + YOH + cue	
	Sex and hormonal differences	WIN	SA and cue (light and sound)	N/T	Reinstatement of drug‐seeking: F > OVX > M	[58]^a^
					Cue‐paired reinstatement: F > OVX > M	
	Sex, reinstatement and attenuation treatment differences	COC and CAF	SA + reinstatement modulator (PRO, CAF and ATO)	PRO and ATO	CAF‐primed reinstatement: F > M	[67]^a^
					Combined treatment for reinstatement attenuation: M + ATO + PRO > F + ATO + PRO	
					Monotreatment for reinstatement attenuation: F + PRO > M + PRO	

Hormonal differences	FEN	SA	E	Cue‐paired reinstatement: OVX + E > OVX	[62]^a^

Abbreviations: ALLO: allopregnanolone, AM251: inverse agonist of Type 1 cannabinoid receptors, ATO: atomoxetine, CAF: caffeine, CBI: cannabinoid 1, CB1R: Type 1 cannabinoid receptors, CIE: chronic intermittent ethanol, COC: cocaine, CP55940: cannabinoid 1 agonist, CPA: conditioned place avoidance, CPP: conditioned place preference, E: oestrogen, EI: estrone, E2: estradiol, EtOH: ethanol, F: female, FA: forced abstinence, FEN: fentanyl, HER: heroin, IntA: intermittent access, M: male, MA: methamphetamine, MIF: mifepristone, NIC: nicotine, N/T: no treatment, OVX: ovariectomized, OXY: oxycodone, PRO: progesterone, RAL: raloxifene, SA: self‐administration, TAM: tamoxifen, THC: tetrahydrocannabinol, WIN: CB1 receptor agonist WIN55,212‐2, YOH: yohimbine.

^a^ More than one phase studied.

^b^ More than one phase studied, including observational experiments described in Table 1.

The primary focus of these assessments was to compare addictive behaviour parameters (i.e., locomotor activation, rewarding effect, increased seeking and consumption) across different addiction phases. Of the 46 selected studies, 18 evaluated sexual comparison alone and eight analysed hormonal effects exclusively (i.e., circulating ovarian hormone profile by exogenous treatment or estrus cycle phase identification). Notably, 10 studies simultaneously assessed both sexual comparisons and hormonal effects on addictive behaviour. Finally, 10 studies addressed simultaneous comparisons between sex and other variables, such as age, model of administration or strain.

**FIGURE 2:**
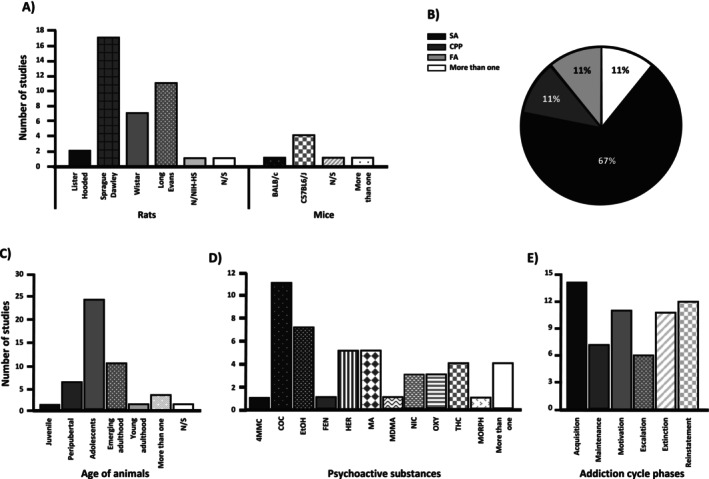
Graphical description of quantitative aspects of the reviewed literature. The studies were classified according to the following characteristics: (A) model of drug administration: forced administration (FA), self‐administration (SA), conditioned place preference (CPP) or use of more than one model; (B) strain and type of rodent used for the studies; (C) life stage of the animals considered for the studies; and (D) type of main comparison of the studies: sex differences, sex and hormonal differences, sex and other types of comparison and hormonal differences. Abbreviations: 4‐MMC: 4‐methylephedrone, COC: cocaine, EtOH: ethanol, FEN: fentanyl, HER: heroin, MA: methamphetamine, MDMA: 4‐methylenedioxymethamphetamine, NIC: nicotine, MORPH: morphine, THC: tetrahydrocannabinol, OXY: oxycodone.

Of the 46 in vivo models, 84.78% involved rats, while 15.21% were murine models. The primary rat strain under investigation was the Sprague Dawley, featured in 17 studies, followed by the Long Evans, examined in 11 studies. Among studies involving mice, the C57BL6/J strain was the most frequently utilised, referenced in five studies (Figure 2A).

The method of PSA administration is chosen based on the biological parameter being evaluated. To assess pharmacological effects, forced administration is used. In contrast, to analyse motivation based on rewarding effects and drug‐seeking behaviour influenced by environmental stimuli, the conditioned place preference (CPP) paradigm is employed. Accordingly, Figure 2B shows that 63% of studies utilised PAS self‐administration, 11% used CPP protocols, 11% used forced administration and 11% used a combination of two or more types of administration.

The age groups of the biological models varied in the selected studies, with adolescents being the most used stage in 24 articles, followed by emerging adulthood and peripubertal groups (each featured in 10 and six articles, respectively). In contrast, the juvenile and young adulthood stages appeared in only one study [36]. Additionally, in four articles, authors used two different ages simultaneously [27, 37, 41, 61], and one article did not mention the life stage nor the approximate weight of the rodents [25]. Other stages of life, including neonatal, infantile, middle adulthood, older adulthood and late adulthood, were notably absent from the studies (Figure 2C).

#### 3.2.1 Experimental Substances

Cocaine (COC) was the predominant experimental substance featured in 11 studies, followed by ethanol (EtOH) (seven studies), heroin (HER) and methamphetamine (MA) (five independent studies for each PAS), natural and synthetic tetrahydrocannabinol (THC) (four studies), oxycodone (OXY) and nicotine (NIC) (three independent studies for each PAS). Less frequently studied substances, including 4‐methylephedrone (4MMC), fentanyl (FEN), 4‐methylenedioxymethamphetamine (MDMA) and morphine (MORPH), were independently featured in only one study (Figure 2D). Notably, in two studies, more than one PAS was used: Wiley et al. used COC, ketamine (KET), MDMA and THC [27]; meanwhile, Lacy et al. used COC and HER [47].

#### 3.2.2 Phases of the Addictive Cycle

Among the 46 analysed studies, most (36 articles) focused on a single phase of addiction; however, 11 studies investigated multiple phases: eight observational and three interventional.

The acquisition phase was the most studied in 14 articles, describing 15 experiments across nine observational and six interventional articles, followed by 12 articles on the reinstatement phase, with an equal distribution between observational and interventional studies (six each). The motivation and extinction phases were independently studied in seven observational articles and four interventional articles (each phase), with Towers et al. being the only interventional study that investigated both phases [62]. The least studied phases were maintenance and escalation, with seven (five observational and two interventional articles) and six (four observational and two interventional articles) articles, respectively (Figure 2E).

#### 3.2.3 Effects of Ovarian Hormones on the Phases of Addiction

##### 3.2.3.1 Acquisition

The observational approach showed that greater addictive behaviour in females was consistently reported when compared to males across various PAS consumption, except for one study. Henricks et al. explored the impact of ovarian hormones on alcohol consumption, finding that females in diestrus had significantly higher intake compared to males, while males and females in estrus showed equivalent alcohol consumption rates [25]. One study found comparable rates of HER consumption between sexes [31]. Another study reported greater sensibility to psychedelics at the onset of the addictive cycle in females when compared to males using the C57BL6L strain [38]. Further sex differences were observed in the redistribution of opioid receptors (ORs) in hippocampal neurons following OXY injections, which decrease excitability and plasticity in males but not in females during estrus [28]. Another aspect evaluated is comparing young versus adult females under a behavioural sensitization model. While no differences were observed with COC, KET or THC, changes were noted with MDMA, where young females did not exhibit behavioural sensitization [27].

Above the six interventional studies that primarily evaluated hormone‐like comparisons in the addictive cycle during the acquisition phase, two studies reported higher PAS consumption in ovariectomized (OVX) females when E2 was administered [55, 57]. Additionally, the introduction of a selective oestrogen receptor (ER) modulator (i.e., tamoxifen [TAM]) significantly heightened NIC consumption [54]. However, one study found a reduction in HER consumption by females after E2 application, with this protective effect reversed upon applying the ER modulator raloxifene [56]. Regarding oral OXY consumption, a higher intake was observed in OVX females in contrast to those females whose gonads were not removed, while in males, the opposite effect was observed: animals whose gonads were not removed presented a lower consumption of OXY [33]. Moreover, in a separate study, Maher et al. showed intact females had higher NIC consumption than OVX females treated with E2 and estrone [57].

##### 3.2.3.2 Maintenance

For the maintenance phase, five observational research articles were examined. Three of these studies identified greater PAS consumption in females compared to males, with one study reporting equivalent COC‐seeking rates between sexes [34]. However, another study found that females during proestrus/estrus exhibited greater seeking behaviour than females in diestrus (Diestrus I and II) when hormone‐based comparisons were implemented [35]. Another study reported greater seeking behaviour in females than males when the C57BL6L strain was used but not with the 129S6/SvEv strain, where the effect was equivalent between sexes [38].

Two interventional studies investigated the maintenance phase, both consistently reporting greater THC seeking in intact females compared to OVX females [58, 59]. Additionally, both studies found that OVX were more sensitive to the substance than males, regardless of the substance's source (natural or synthetic). Moreover, Fattore et al. reported greater PAS seeking in Lister Hooded rats [58].

##### 3.2.3.3 Motivation

Seven observational studies focused on the motivation phase, with six consistently reporting higher levels of behavioural motivation for PAS consumption among females than males. Two studies found no differences between sexes: Cullity et al. found no differences in developing MA‐seeking behaviour when CPP was used [41], and Algallal et al. found no differences in COC intake [45]. Nonetheless, in the latter study, the long‐access self‐administration protocol was more effective in producing sex differences in COC consumption, with female rats consuming more COC than male rats. In contrast, the intermittent access protocol was more effective in producing sex differences in sensitization‐related changes, which were more pronounced in females. Additionally, the rewarding effects of 4MMC were higher in females under the social‐CPP protocol compared to CPP alone [42].

All interventional studies focused on the same phase, using exogenous E2 treatments to identify sensitivity to COC, MA and FEN in hormone‐supplied females [39, 60, 61, 62]. Furthermore, Torres et al. demonstrated that intact females displayed enhanced CPP produced by 1.0 g/kg of EtOH [61]. This suggests that ovarian hormones likely mediate the rewarding effects of EtOH, as OVX females did not exhibit a drug‐seeking behaviour. In addition, the study found no significant differences in aversion to EtOH among intact females tested across different phases of the estrus cycle, OVX females and males.

##### 3.2.3.4 Escalation

In three studies modelling the escalation phase (observational approach), females exhibited greater consumption and a higher tendency to escalate PAS consumption than males. One study found higher COC consumption in females in estrus when a male partner was present and lower HER intake in females in proestrus (regardless of the social context); in both cases, PAS consumption was higher in females than in males [47]. Another study reported greater MA consumption under the long‐access versus short‐access model [48]. Only one of the studies reported higher MA consumption in males than females [49].

Two interventional studies modelled the escalation phase, yielding conflicting results. Satta et al. reported that alcohol consumption in intact females was higher than in OVX females, while males consumed less than both female groups [63]. Additionally, E application in OVX females increased their consumption rate compared to untreated females. In another study, no sexual differences were observed in COC consumption, but COC and remifentanil intake increased during the estrus phase [64].

##### 3.2.3.5 Extinction

Among the seven observational studies that modelled extinction, two reported greater seeking behaviour by females than males, specifically for COC^53^ and HER [31]. In Bossert et al., hormonal comparisons revealed that HER consumption was independent of the estrus cycle [50]. Another two studies found similar effects between sexes when MA was used [48, 49]. One more study was conducted in different age groups, and no sex differences were found regarding MORPH intake. Nevertheless, young male adults were significantly more susceptible than females to the aversive effects of withdrawal: symptoms decreased by 7 days in males and 3 days in females [51]. Sutton et al. discovered an interesting finding during the extinction phase: the implementation of a system of random alternating punishments combined with a nondrug reinforcer significantly suppressed alcohol‐seeking behaviour in males compared to females [52]. This indicates that females exhibited greater resistance to extinction than males.

Two of the four interventional studies that modelled the extinction phase reported increased COC and FEN consumption when OVX females were administered with exogenous E2. However, E2 did not affect orchiectomized (OCX) males administered with the hormone [62, 65]. Contrastingly, Swalve et al. reported no sex differences in COC intake [67]. In another study, food restriction increased HER seeking in female rats, similar to males. Ovariectomy had no significant effect on HER seeking. However, E2 blocked the increase in HER seeking caused by food restriction, while PRO had no effect on HER seeking in either food‐restricted or sated rats [66].

##### 3.2.3.6 Reinstatement

Six studies assessed the reinstatement phase using an observational approach. In five of these studies, higher relapse rates were found in females [32, 46, 48, 50, 52]. One study reported that reinstatement of orally administered OXY was equal between the sexes [33].

In five of six interventional studies where stressors such as yohimbine (YOH), caffeine (CAF) or cues were paired with reinstatement, the relapse rate was higher in females than in males [34, 58, 67, 68, 69]. Further experimental studies examining the attenuation of reinstatement revealed that PRO significantly reduced COC intake in females but not in males [67]. Finally, in a study involving exclusively OVX females, greater reinstatement of FEN intake was reported in the group receiving exogenous E2 compared to those receiving vehicle alone [62].

### 3.3 Mechanisms Underlying Ovarian Hormone Modulation on Addictive Behaviour

Most reviewed studies did not conduct additional experiments to elucidate the neurochemical mechanisms responsible for observed behavioural outcomes. Proposed mechanisms are largely speculative, relying on literature identified by the authors of experimental studies (Table 3).

**TABLE 3:** Neurochemical mechanisms of response to psychoactive substances (PASs) influenced by ovarian hormones.

Modelled addiction phase	PAS	Outcome	Referring to the group marked in bold vs. those not highlighted				Reference
			PRO level relative to other groups	ES level relative to other groups	Drug‐related outcome	Proposed mechanism	
Acquisition	COC	OVX + E > OVX + ES + AM251	—	↑	↑	Increased sensitivity due to the estradiol‐mediated activation of mGluR5, which in turn promotes neuroplasticity in the reward circuits	[53]
	COC, KET, MDMA andTHC	FAdult + COC = FAdoles + COC; FAdult + KET = FAdoles + KET; FAdoles + MDMA > FAdult + MDMA; FAdoles + THC = FAdult + THC	↑	↑	↑	Enhanced rewarding effects of KET by acting as NDMA blockers leading to neuroadaptations with potential addiction liability	[27]
	NIC	F + TAM > F	—	↑	↑	CPP facilitated by TAM due to E antagonism and PKC inhibition; however, an E agonist effect of TAM on reward mechanisms cannot be ruled out	[54]
	CP55940 (CB1 agonist)	OVX + E > F	↓	↑	↑	Modulation of dopaminergic reward due to sensitivity in CB1R density and function to ovarian hormones	[55]
	HER	Proestrus + RAL > Proestrus + MIF	↑	↓	↑	Effect of E in increased endogenous opioid receptor tone (met‐encephalin), upregulation of proenkephalin‐A mRNA and mu receptor mRNA, as well as greater mu stimulation of GTPγS binging in the dorsal striatum, leading to reduced opioid intake	[56]
		OVX + PRO > OVX + E	↑	↓	↑		
	NIC	F > OVX F > OVX + E1 F > OVX + E1 + E2	—	↑	↑	Greater rewarding effects. High E1 from intact females augments α7nAChR and ERα mRNA, which in turn results in a blockade of DA firing, yet such effects are suppressed by NIC. As such, DA blockade is ceased, instead occurs an increase in dopaminergic activity of the VTA and enhanced DA release in the MSNs of the NAcore	[57]
	OXY	OVX > F‐sham	—	↓	↓	An undescribed effect between E and hunger is suggested to play in the downregulation of OXY consumption, which is reversed by OVX	[33]
		M‐sham > OCX	—	—	—	An unclear testosterone‐dependent mechanism of OXY consumption is proposed	
Maintenance	COC	Estrus > diestrus	↓	↑	↑	Effect of ES in increased activity of VTA dopaminergic neurons following Thr53 phosphorylation, enhanced ability of COC to inhibit DAT and greater rewarding properties	[35]
	WIN (CB1 agonist)	F > OVX > M	↑	↑	↑	Influence of ovarian hormones on enhanced rewarding properties, such as endogenous cannabinoids and modulation of drug‐seeking behaviour	[58]
	THC	F > OVX	—	—	↑	Greater rewarding effects due to increased CB1 receptors in the striatum and enhanced GTPγS coupling	[59]
Motivation	COC	No‐estrus > estrus	↑	↓	↓	Possible role of PRO in reducing anxiety‐like behaviour and COC preference, contrary to E, related to increased cocaine‐induced reward	
	COC	H > OVX; OVX + E > OVX	↓	↑	↑	E‐dependent modulation of DA firing and transmission in the mesolimbic system, as well as D1R calcium/calmodulin‐dependent protein kinase II activity	[39]
	MA	OVX + E > OVX	↓	↑	↑	Greater E‐induced locomotor sensitization due to modifications in DA release in various measures of the mesocorticolimbic DA system and areas such as the NAc	[60]
	EtOH	FAdult > AdultOVX FAdoles > MAdoles	↑	↑	↑	Increased rewarding effect by an E2‐induced normalization of EtOH‐promoted DA release in the PFC	[61]
		Proestrus > no‐proestrus	↓	↑	↑		
	FEN	OVX + E > OVX	—	↑	↑	A possible nondetailed modulatory effect of E on the mesolimbic dopaminergic reward system is suggested	[62]
Escalation	COC	Estrus > proestrus	↓	↑	↑	ES‐dependent effect on reward enhancement	[47]
	HER	No‐estrus > proestrus	↑	↓	↑	Attenuation by PRO anxiolytic‐like treatment	
	EtOH	OVX + E > OVX	—	↑	↑	—	[63]
	COC + REMIFEN	Estrus > meta/diestrus	↓	↑	↑	Increased subjective drug effects when oestrogen levels are high contributed to high behavioural economic demand of COC by prior opioid consumption (REMIFEN)	[64]
Extinction	HER	Proestrus = no‐proestrus	—	—	=	No‐estrus cycle‐dependent effect	[31]
	COC	OVX + E > ORQ + E	—	↑	↑	Enhanced ES‐induced sensitization due to increase in DA from the DLS in F	[65]
	HER	F + PRO > F + E	↑	↓	↑	No proposed mechanism but stress related by food restriction	[66]
	FEN	OVX + E > OVX	—	↑	↑	ES‐dependent effect is proposed through unspecified mechanisms in the influence of synaptic plasticity enhancing the long‐term effects of extinction	[62]
Reinstatement	COC	F + YOH > F + YOH + ALLO; M + YOH + ALLO = M + YOH	—	↑	↑	Sex‐specific action of ALLO due to anxiolytic effects on the HPA axis and reduction of COC craving	[34]
	COC	Proestrus + YOH + cue > no‐proestrus + YOH + cue	↓	↑	↑	Greater reinstatement due to elevated ACTH and corticosterone levels due to YOH and the phase of the estrus cycle. An increase in striatal DA transport and uptake sites due to ES action is also mentioned	[69]
	WIN	WIN: F > OVX > M	↑	↑	↑	Facilitated rewarding properties of cannabinoid agonists by undescribed ovarian hormone‐dependent mechanisms	[58]
		WIN + cue: F > OVX > M	↑	↑	↑		
	COC and CAF	Combined treatment for attenuation: F + ATO + PRO = M + ATO + PRO.	↑	↓	=	Uncharacterized mixed effects in ATO treatment	[67]
		Monotreatment for attenuation: F + PRO > M + PRO	↑	↓	↓	Anxiolytic effect of PRO through specific undescribed mechanisms	
	FEN	OVX + E > OVX	—	↑	↑	Increased sensitivity to drug‐seeking through undescribed E dependent mechanisms	[62]

Abbreviations: ACTH: adrenocorticotropic hormone, ALLO: allopregnanolone, AM251: Type 1 cannabinoid receptor inverse agonist N (piperidin‐1‐yl)‐5‐(4‐iodophenyl)‐1‐(2,4‐dichlorophenyl)‐4‐methyl‐1H‐pyrazole‐3carboxamide, ATO: atomoxetine, CAF: caffeine, CBI: cannabinoid 1, CB1R: Type 1 cannabinoid receptors, COC: cocaine, DA: dopamine, DAT: dopamine transporter, D1R: dopamine receptor, DLS: dorsolateral striatum, E: oestrogen, EI: estrone, E2: estradiol, ERα: alpha oestrogen receptor, EtOH: ethanol, F: female, FA: forced abstinence, FEN: fentanyl, GTPγS: medium spiny neurons, HER: heroin, HPA: hypothalamic–pituitary–adrenal, KET: ketamine, M: male, MA: methamphetamine, MDMA: 4‐methylenedioxymethamphetamine, MIF: mifepristone, mGluR: metabotropic glutamate receptors, mRNA: messenger RNA, MSNs: medium spiny neurons, NAc: nucleus accumbens, nAChR: nicotinic acetylcholine receptors, NAcore: nucleus accumbens core, NIC: nicotine, NMDA: N‐methyl‐D‐aspartate, OVX: ovariectomized, OXY: oxycodone, PFC: prefrontal cortex, PKC: protein kinase C, PRO: progesterone, RAL: raloxifene, TAM: tamoxifen, THC: tetrahydrocannabinol, Thr53: threonine 53, VTA: ventral tegmental area, WIN: CB1 receptor agonist WIN55,212‐2, YOH: yohimbine.

A consistent finding across several studies reviewed was that E2, whether naturally elevated or administered exogenously, increases the consumption of specific PAS. These substances included KET, COC, MA, NIC and THC. However, HER was unique in that high plasma E levels were associated with lower consumption during the acquisition phase [56]. Interestingly, higher HER consumption was correlated with elevated plasma PRO levels during the extinction phase [66].

The proposed mechanisms for the link between elevated plasma E2 levels and a more robust response to seek and/or consume PAS coincided in promoting greater rewarding effects in the first four phases of addiction. Wiley et al. reported high consumption of KET in young females because of the substance's ability to act as an NMDA channel blocker, with the capacity to induce neuroadaptations with addiction liability [27]. The signalling pathways involved included the antagonism of ER and the inhibition of PKC signalling. TAM, the selective ER modulator, was shown to promote catecholamine release, which contributed to higher NIC consumption in females [54].

**FIGURE 3:**
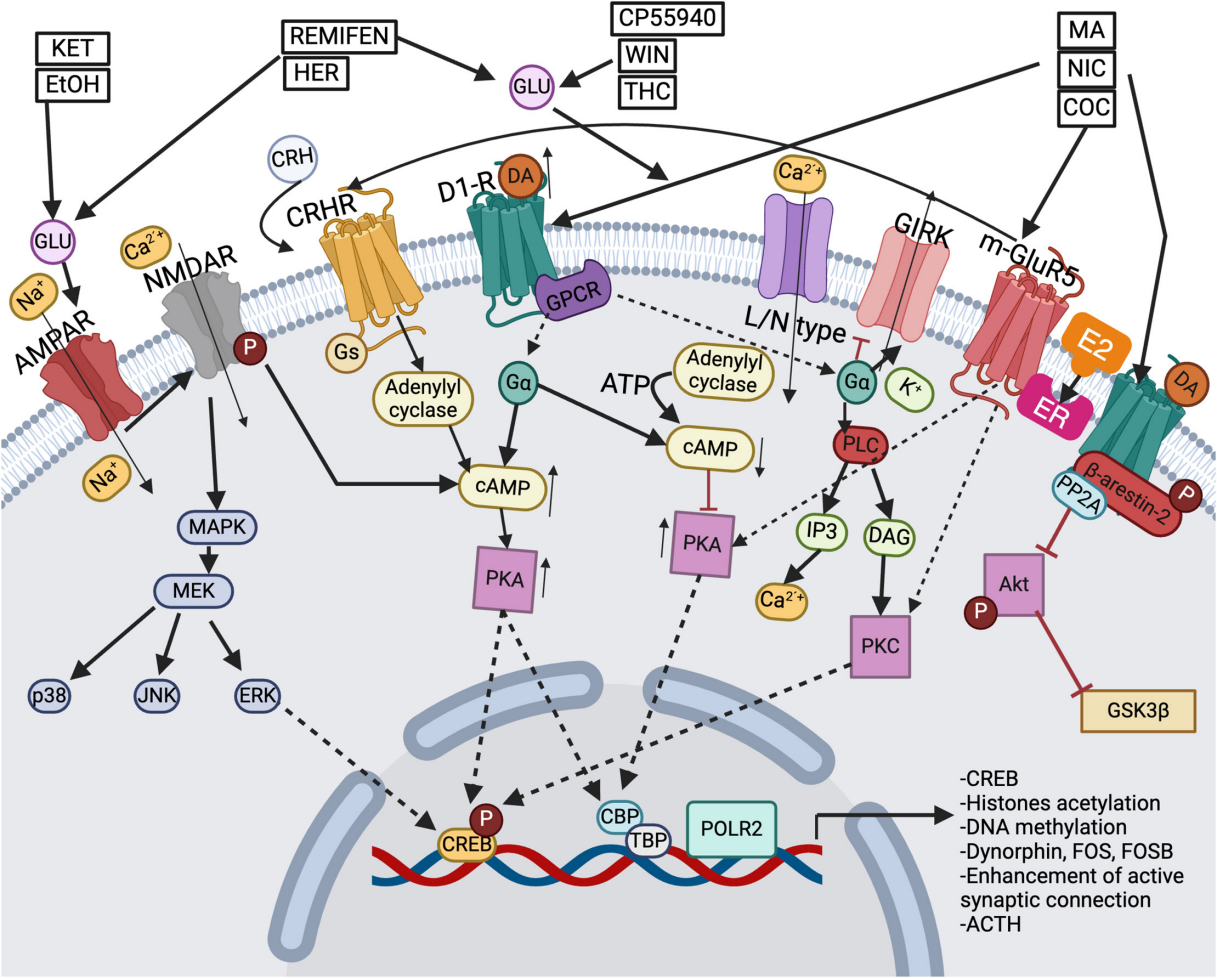
Schematic representation of the dopaminergic reward signalling pathways and their interaction with oestrogen signalling. PAS compounds (COC, MA and NIC) competitively inhibit DAT upon reaching the brain, thereby preventing neuronal reuptake of DA and promoting its synaptic accumulation. D1 and D2 receptors are associated with G proteins; D1‐associated subunits promote signal exchange with other pathways such as MAPK‐MEPK‐ERK and CREB, a gene regulator potentially involved in the synaptic remodelling of receptor desensitization and enhancing the memory effects of E2 in response to membrane‐initiated signalling events. Furthermore, PAS (Ket, EtOH, Her, Remifen, THC, Win and CP5594) can modulate glutamate release, directly or indirectly, through D1‐R activation, leading to an increase in AMPAR and NMDA receptor trafficking. Alongside, the neurosteroid E2 binds to its receptor and other transmembrane receptors (such as metabotropic glutamatergic receptor ‘mGLUR5’) and initiates kinase signalling cascades that lead to additional upregulatory processes, such as protein synthesis and NMDA channel phosphorylation; all of which contribute to modulations in synaptic function, neuronal plasticity and glutamatergic transmission. Elevated ACTH and corticosterone during proestrus (high‐oestrogen phase) further interact with DA pathways, amplifying synaptic modulation. Abbreviations: ACTH: adrenocorticotropic hormone, AMPAR: α‐amino‐3‐hydroxy‐5‐methyl‐4‐isoxazolepropionic acid receptor, COC: cocaine, CP55940: cannabinoid 1 agonist, CREB: cAMP response element‐binding, D1R: dopamine receptor, DA: dopamine, DAT: dopamine transporters, E2: estrone, ERK: extracellular‐signal‐regulated kinase, EtOH: ethanol, HER: heroin, Ket: ketamine, MA: methamphetamine, MAPK: mitogen‐activated protein kinases, NMDA: N‐methyl‐D‐aspartate, NIC: nicotine, FA: forced administration, PASs: psychoactive substances, SA: self‐administration, THC: tetrahydrocannabinol, WIN: CB1 receptor agonist WIN55,212‐2. Figure modelled after [70, 71]. Created with BioRender.com.

**FIGURE 4:**
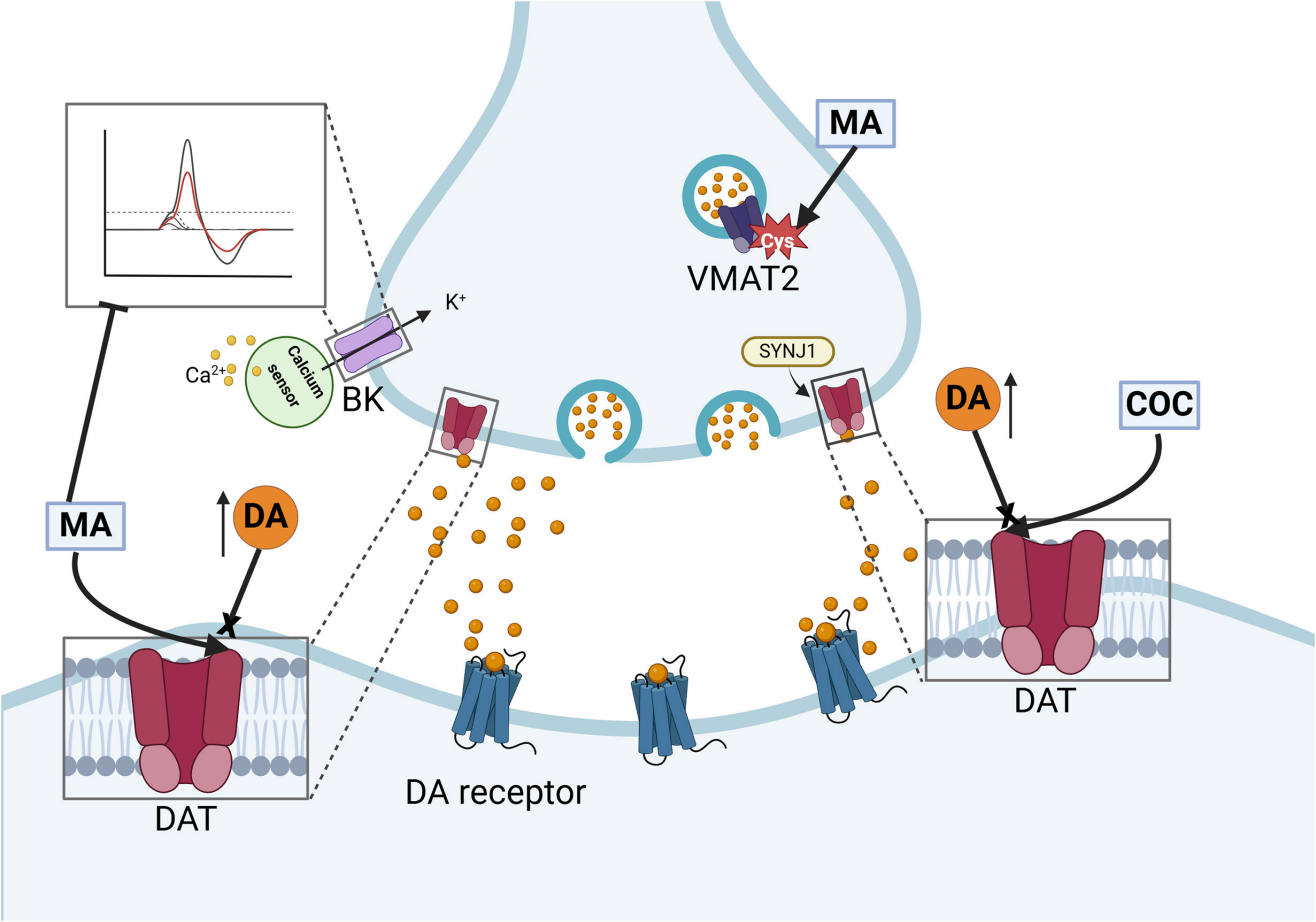
Mechanisms of methamphetamine and cocaine on dopamine transport and neuronal excitability. MA is the substrate for DAT, which competes with DA and causes increased DA extracellular, while it alters neuronal excitability by reducing the amplitude of calcium‐activated potassium currents (BK channels). In addition, MA modulates DA release through activation of σ1R receptors in the endoplasmic reticulum of cells of the NAc, impairing the function of the VMAT2 via oxidation of a cysteine residue. On the other hand, COC inhibits DA reuptake by blocking DAT in its outward‐facing conformation and inducing its intracellular translocation, mediated by SYNJ1. Abbreviations: ACTH: adrenocorticotropic hormone, AMPAR: α‐amino‐3‐hydroxy‐5‐methyl‐4‐isoxazolepropionic acid receptor, COC: cocaine, CP55940: cannabinoid 1 agonist, CREB: cAMP response element‐binding, CRH: corticotropin‐releasing hormone, CRHR: corticotropin‐releasing hormone receptor, D1R: dopamine receptor, DA: dopamine, DAG: diacylglycerol, DAT: dopamine transporters, E2: estrone, ER: oestrogen receptor, ERK: extracellular‐signal‐regulated kinase, EtOH: ethanol, FA: forced administration, GLU: glutamate, HER: heroin, IP3: 1,4,5‐trisphosphate, Ket: ketamine, m‐GluR5: metabotropic glutamate receptor 5, MA: methamphetamine, MAPK: mitogen‐activated protein kinases, NMDA: N‐methyl‐D‐aspartate, NIC: nicotine, PASs: psychoactive substances, PCL: phospholipase C, SA: self‐administration, SYNJ1: synaptojanin‐1, THC: tetrahydrocannabinol, VMAT2: vesicular monoamine transporter 2, WIN: CB1 receptor agonist WIN55,212‐2. Figure modelled after [72, 73, 74]. Created with BioRender.com.

In an independent investigation by Maher et al., it was postulated that the molecular mechanism driving heightened NIC consumption among intact females stems from elevated dopaminergic activity within the ventral tegmental area (VTA) and nucleus accumbens core (NAcore) [57]. This phenomenon is attributed to NIC's blockade of the natural inhibitory influence of E2 on dopamine (DA) release, thereby leading to increased stimulation (Figure 3). However, MA and COC have different effects on dopamine transporter (DAT), briefly related to MA, which acts as a substrate for the DAT, competing with DA and causing sustained increases in extracellular DA while altering neuronal excitability by reducing the amplitude of calcium‐activated potassium currents (BK channels), which in turn increases extracellular DA levels. In the NAc, MA modulates DA release through activation of σ1R receptors in the endoplasmic reticulum, impairing the function of the vesicular monoamine transporter 2 (VMAT2) via the oxidation of a cysteine residue. COC inhibits DA reuptake by blocking DAT in its outward‐facing conformation and inducing its intracellular translocation, a process mediated by synaptojanin‐1 (SYNJ1) [72, 73, 74] (see Figure 4).

Several proposed mechanisms have suggested E2‐mediated alterations in DA firing and reuptake, particularly in neurons exhibiting activity within the VTA and NAcore [35, 39, 47, 57, 59, 60, 61, 65, 69]. Figure 3 illustrates evidence suggesting higher Type 1 cannabinoid receptor (CB1R) density in females positively modulates dopaminergic reward [55, 59]. This effect is further amplified by increased activation of metabotropic glutamate receptors (mGluR5) [53] and enhanced endocannabinoid release [43], all of which are influenced by E2.

**FIGURE 5:**
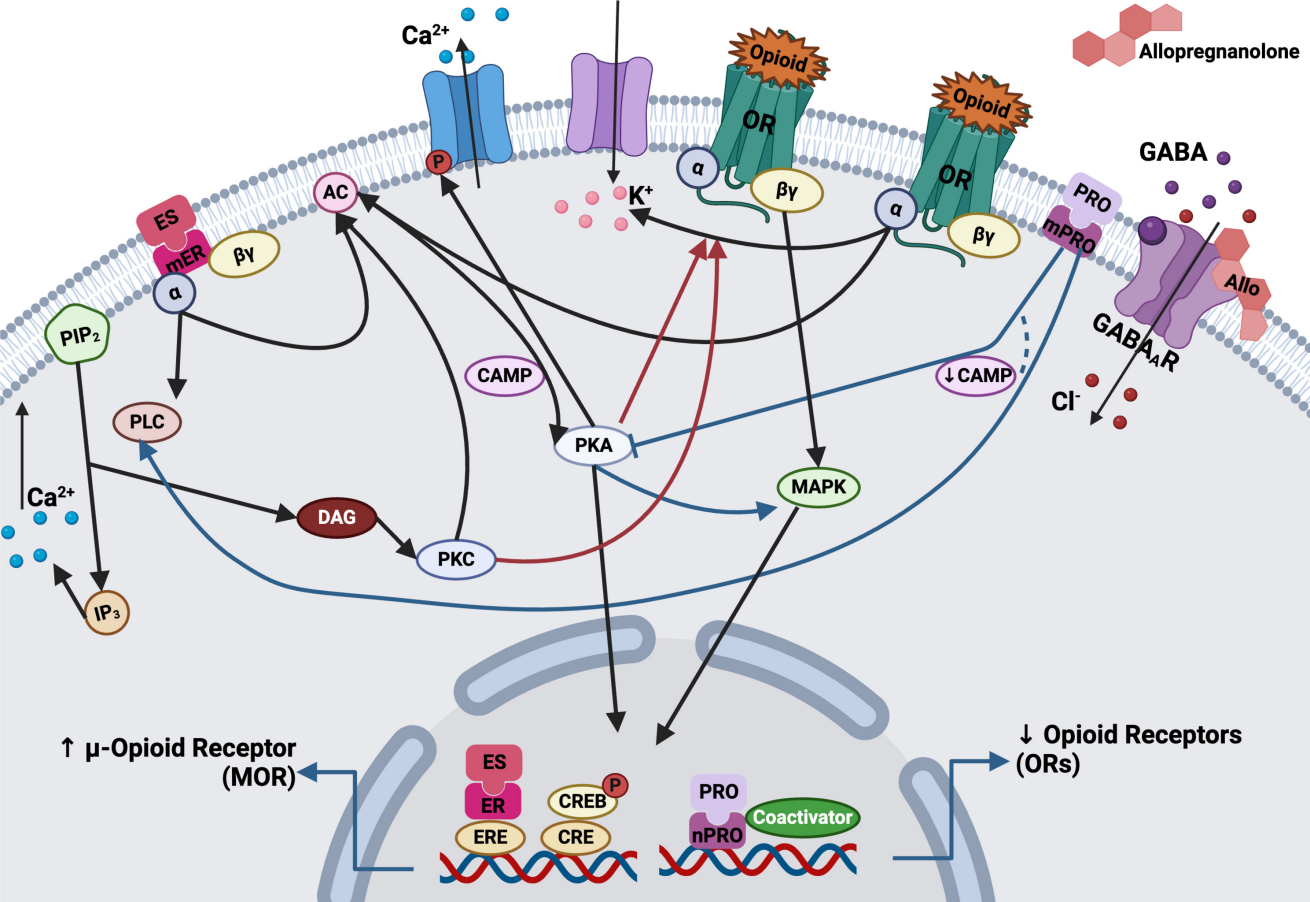
Postulated interaction among oestrogen, progesterone and opioid receptors. Estradiol (ES), when binding to its mER, can modify ionotropic conductance by promoting the phosphorylation of ionotropic receptors and the decoupling of OR from their ion channels. It also triggers intracellular signalling interactions via the G protein subunits (α, β and γ) to which it is coupled. The activation of PLC catalyses the conversion of membrane‐bound PIP2 into IP3 and DAG. Subsequently, IP3 promotes the release of Ca+, starting calcium‐dependent signalling. DAG activates PKC, which in turn activates AC, increases cAMP and can phosphorylate ion channels and cAMP response CREB. ES can also bind to nuclear RE dimers, such as the ERE in DNA, promoting the transcription of specific genes, including the upregulation of μ‐opioid receptors (MOR) and leading to reduced opioid intake. PRO binds to its mPRO and can alter gene transcription regulated by second messengers (cAMP/PKA and Ca+/PKC) and signal transduction pathways MAPK, resulting in the phosphorylation of nuclear transcription factors. PRO can also bind to its nuclear receptor, contributing to changes in gene transcription, such as the downregulation of ORs. Additionally, ALLO modulates the activity of synaptic GABA_A_ receptors, facilitating chloride ion (Cl^−^) entry and regulating neuronal excitability. Abbreviations: ALLO: allopregnanolone, AC: adenylate cyclase, CREB: element‐binding proteins, ERE: oestrogen response element, ES: oestrogen, GABA: gamma‐aminobutyric acid, GABA_A_R: GABA_A_ receptor, IP3: inositol 1,4,5‐trisphosphate, mER: membrane oestrogen receptor, MAPK: mitogen‐activated protein kinases, mPRO: progesterone membrane receptor, ORs: opioid receptors, PCL: phospholipase C, PKC: protein kinase C, PIP2: phosphatidylinositol 4,5‐bisphosphate, PRO: progesterone. Figure modelled after [75, 76]. Created with BioRender.com.

The ameliorative effects observed after administering PRO and allopregnanolone (ALLO) were primarily attributed to their unspecified anxiolytic properties. These hormones were suggested to mitigate cravings for PAS during the extinction and reinstatement processes. Consequently, it was hypothesized that such anxiolytic effects would lead to a reduction in PAS consumption and/or seeking behaviour [34, 39, 47, 66, 67]. This hypothesis is further supported by Feltenstein et al. who reported that an α2 norepinephrine (NE) receptor antagonist increased anxiety and intake [69]. Despite these findings, the molecular mechanisms underlying the effects of PRO and its metabolites in rodent models remain largely unexplored. While Smith et al. demonstrated that PRO did not significantly decrease HER intake, E administration was associated with a reduction in its consumption [56]. This interaction was hypothesized to be facilitated by an ovarian hormone‐induced upregulation of endogenous OR tone, resulting in a protective effect on opioid consumption (Figure 5).

### 3.4 Quality and Risk of Bias Assessment

**FIGURE 6:**
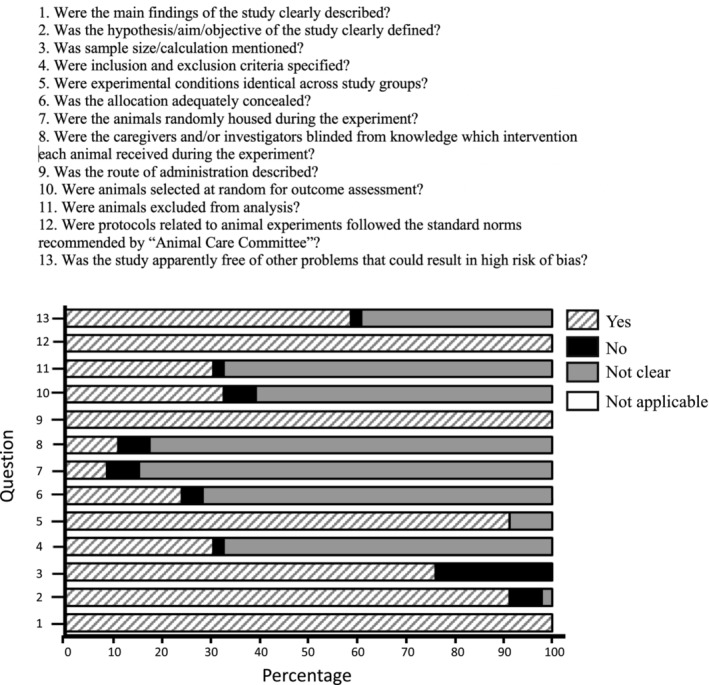
Evaluation of the methodological quality and assessment of the risk of bias. The bars represent the percentage of the articles found in each category.

The risk assessment was made using the instrument modified from Hooijmans et al., OHAT and Recommendations for Ensuring Good Scientific Inquiry tools [22, 23, 24]. The highest bias rate was identified in Question 8: ‘Were the researchers blinded to knowledge about the experimental group?’ Only 10.9% of studies reported blinding in study design, 6.5% denied impartiality in this aspect and the remaining 82.6% were imprecise. The criteria pertaining to the random allocation of methodological domains, as addressed in Question 10 (“Were the animals selected at random outcome assessment?’), was considered within a threshold of low bias risk. It was found that 60.9% of the studies had unclear scores. However, in 32.6% of the studies, the process of randomization for outcome assessment was reported, while in 6.5% of the studies, this randomization process was reportedly absent. Notably, the domain demonstrating the lowest bias risk within this instrument corresponded to the first question of the tool: ‘Were the main findings of the study clearly described?’ with all studies receiving affirmative responses (Figure 6).

## 4 Discussion

### 4.1 Overview of Ovarian Hormones Effects on PAS Consumption

Epidemiological surveys [77] and clinical reports indicate that addiction prevalence is generally higher in men than in women, leading research to primarily focus on men. However, recent studies have highlighted significant gender differences in binge behaviours and the progression of drug use. For example, women tend to initiate drug use at an earlier age, develop substance use disorder more rapidly and seek treatment sooner than men [78, 79, 80]. These findings accentuate the need for a deeper understanding of both behavioural and cellular mechanisms in addiction.

This systematic review explored the influence of ovarian hormones on various phases of the addictive cycle in female murine models, emphasizing their association with PAS consumption based on recent findings. Among the 46 studies reviewed, a key insight was the role of E2—both endogenously elevated and exogenously administered—increasing the consumption of substances such as KET, COC, MA, NIC and THC.

As observed in rodents, female rhesus monkeys acquired oral phencyclidine self‐administration more rapidly than males, highlighting their faster acquisition of drug‐reinforced behaviour [81]. Notably, while E2 modulated HER‐seeking behaviour under food restriction, PRO did not exert a significant effect [66]. This contrasts with its role in COC use, where PRO demonstrated a protective effect by attenuating relapse, underscoring its therapeutic potential in reducing relapse rates among rodent females [67]. Moreover, elevated E2 levels during acquisition mitigated the PRO‐driven increase in HER use [56], suggesting a nuanced interaction between these hormones. However, as Nicolas et al. indicated, the protective effect of E2 observed in Sedki et al.'s study suggests that this hypothesis may not generalize to opioids, underscoring the need for further research on substance‐specific hormonal influences in addiction [66, 82].

PRO's role in reducing drug consumption, particularly during the luteal phase, suggests potential therapeutic applications [83, 84]. Timing interventions to coincide with the luteal phase could reduce cravings and improve outcomes. Additionally, PRO's protective effects during pregnancy and its role in smoking cessation [85] further support its potential as a treatment target. Tailoring relapse prevention programs to menstrual phases may enhance success rates, with women in the luteal phase less prone to relapse [86].

### 4.2 Methodological Influences in Assessing Hormonal Influences in Addiction Research

Evidence from various models used to study addiction, including humans [87, 88, 89, 90], non‐human primates [91, 92, 93, 94, 95, 96, 97] and rodents, demonstrates that sex differences in drug‐seeking behaviours are influenced by factors such as the specific substance, experimental paradigm, strain, age and addiction phases. This emphasizes the critical role of genetic background and methodology in addiction research.

A consistent trend observed in the reviewed articles was that younger female rodents appeared to be more vulnerable to addiction than adults, whereas older individuals showed a reduction in PAS consumption [27, 41, 51, 61]. These findings are supported by Juárez‐Portilla et al., which underscore adolescence as a critical period for initiating drug consumption and highlight its long‐term implications in humans [98]. The heightened vulnerability of younger females to addiction may be attributed to the unique neural plasticity and hormonal changes during this stage of development. Adolescents are particularly susceptible to the long‐term cognitive and behavioural impacts of substance use, as drugs target neural mechanisms critical for academic and social development [98]. In contrast, the observed decline in PAS consumption among older individuals may reflect changes in neurobiology, life priorities or reduced exposure to high‐risk environments with age. The relationship between age and reward sensitivity is well‐documented, with evidence showing that age‐related changes in the reward system, particularly in dopaminergic function, can reduce the intensity of reward responses [99]. These findings underline the importance of early prevention efforts targeting youth, especially females, to mitigate the lifelong consequences of substance abuse.

In terms of genetic background, Jaster et al. found greater seeking behaviour in female C57BL6L mice compared to males, while no sex differences were observed in the 129S6/SvEv strain [38]. Similarly, Bossert et al. reported stronger PAS‐seeking behaviour in Long Evans rats than in Lister Hooded rats, underscoring the impact of strain selection [50].

The method of PAS administration or conditioning profoundly affects addictive behaviours and their underlying neurobiological mechanisms, making protocol‐dependent variations crucial for interpreting addiction studies. Algallal et al. showed that prolonged administration protocols enhanced sex differences in COC consumption, while intermittent protocols were more effective in eliciting sensitization‐related changes [45]. These findings suggest that prolonged exposure amplifies inherent sex differences in drug consumption, whereas intermittent exposure highlights neuroadaptive responses.

Wronikowska et al. found that females experienced greater rewarding effects of low‐dose 4MMC under social‐CPP protocols compared to CPP alone, underscoring the role of social context and associative learning in modulating drug‐seeking behaviours [42]. Ryan et al. observed heightened associative learning in females during OXY‐seeking behaviour, accompanied by increased delta ORs in CA‐3 pyramidal neurons [40]. Ashirova et al. reported that opioid‐seeking behaviour disrupted hippocampal OR redistribution in males but not females, suggesting sex‐specific neuroadaptive responses linked to associative learning in CPP protocols [28].

Cullity et al. noted no sex differences in MA‐seeking behaviour using CPP but observed greater female aversion under conditioned place aversion (CPA), highlighting the differential impact of positive versus negative reinforcement on sex‐specific drug responses [41]. Reichel et al. reported higher MA consumption in long‐access models compared to short‐access models, which better mimic chronic drug exposure in humans, providing insights into addiction escalation processes [48].

### 4.3 Phase‐Specific Hormonal Effects

Each phase of addiction has distinct biological, psychological and social factors influencing behaviour. For instance, neural circuits involved in drug craving during withdrawal differ from those driving the initial pleasurable effects of drug use [100]. Studying these mechanisms in a phase‐specific manner may reveal unique vulnerabilities and risk factors that lead to the development of more effective pharmacological and behavioural therapies. While the acquisition phase identifies individuals at high risk and the reinstatement phase is critical for understanding relapse, both were the most frequently studied phases among the reviewed articles. Only 24% of studies addressed multiple phases: eight covered more than one phase, and Reichel et al. and Towers et al. uniquely examined three phases [46, 48, 62]. Notably, only four studies showed consistent effects of ovarian hormones on PAS intake across phases, underscoring the importance of a comprehensive approach to addiction research for phase‐specific interventions.

### 4.4 Neurobiological Mechanisms

Most studies did not investigate neurochemical mechanisms, relying on existing literature for speculative explanations. Multiple studies supported E2‐mediated changes in DA firing and reuptake, particularly in the VTA and NAcore [17, 35, 39, 47, 59, 60, 61, 65, 69]. Higher CB1R density escalated mGluR5 activation, and enhanced endocannabinoid release in females was linked to E2's positive modulation of dopaminergic reward [53, 55, 58, 59].

E2 modulators, particularly selective oestrogen receptor modulators (SERMs) like TAM and raloxifene, significantly influence addictive behaviours. TAM, exhibiting both ER antagonist and agonist properties depending on the target tissue, increases NIC consumption by antagonizing ER and inhibiting PKC signalling [54]. This alteration in ER activity can enhance dopaminergic signalling, increasing the rewarding effects of NIC [18, 101]. Additionally, NIC's blockade of E2's inhibitory effect on DA release further heightens consumption [57]. E2, on the other hand, reduces HER consumption by modulating the dopaminergic system and interacting with ORs [56]. It enhances DA release in reward‐related brain areas, attenuating HER's reinforcing effects [69]. However, raloxifene, an ER antagonist, reverses this effect, increasing HER consumption and indicating the necessity of functional ER for E2's protective effects.

The molecular mechanisms of PRO and its metabolites, particularly ALLO, involve key interactions with neural systems linked to addiction by reducing PAS consumption and seeking behaviour through their anxiolytic effects acting in the regulation of the hypothalamic–pituitary–adrenal (HPA) axis [34, 39, 47, 66, 67, 69, 102]. PRO acts through membrane (mPRO) and nuclear receptors, activating intracellular signalling pathways such as cAMP/PKA, Ca+/PKC and MAPK. These pathways can regulate gene transcription and influence processes like OR expression [75, 76].

ALLO, a potent positive allosteric modulator of GABA‐A receptors, plays a role in addiction‐related pathways by modulating DA levels in the NA [103] and prefrontal cortex [104]. These regions are critical for reward and executive control. By enhancing GABAergic activity, ALLO can suppress excitatory signals in reward circuits, potentially reducing the reinforcing effects of drugs like COC.

In studies on YOH‐induced reinstatement of COC‐seeking behaviour, ALLO demonstrated notable effects, though females showed greater resistance to the extinction of COC‐seeking behaviour after drug removal [67]. Sensitivity to ALLO appears influenced by PRO levels, with females in the proestrus phase (high PRO) being less responsive to ALLO, suggesting a modulatory role for PRO in the efficacy of its metabolites [103].

Together, this evidence highlights PRO's dual role in modulating neurochemical and genetic factors involved in addiction, emphasizing the need for further research on the interplay between PRO, its metabolites and GABAergic and dopaminergic systems in addiction and recovery.

These findings highlight the importance of hormonal modulation in addiction and the need for further research to elucidate underlying mechanisms.

### 4.5 Circadian and Infradian Rhythms in Addictive Behaviour

Biological oscillations, specifically circadian and infradian rhythms, may contribute to sex differences in addiction. Lynch and Taylor found that female rats exhibited a more dysregulated diurnal pattern of COC self‐administration under extended‐access conditions and demonstrated higher levels of responding during reinstatement testing compared to male rats [105]. Additionally, Guilding and Piggins explored the role of circadian and infradian oscillators in the mammalian brain, highlighting how these rhythms influence neurochemical activities, including dopaminergic signalling [106]. Hood et al. further demonstrated that endogenous DA regulates the rhythm of the clock protein PER2 in the rat dorsal striatum, a process influenced by daily activation of D2 DA receptors and likely modulated by circadian rhythms [100]. E2 has been found to enhance dopaminergic activity in the brain, particularly in regions associated with reward and reinforcement. Notably, E modulates dopaminergic neuron activity in the hypothalamus through cell signalling‐dependent mechanisms [107]. It involves uncoupling μ‐opioid and GABAB receptors from G‐protein‐regulated K+ channels and the subsequent signalling cascade through PLC, PCK, PI3K and PKA [108, 109, 110]. Beyond its direct effects on dopaminergic neuron activity, the role of E in addiction and reward processing is intricately linked to circadian rhythms. For example, a study by Chung et al. demonstrated that the circadian nuclear receptor REV‐ERBα influences midbrain DA production and mood regulation, with E2 levels modulating this interaction [111]. Additionally, research by Blume et al. highlights how hormonal changes, including E2, affect the dopaminergic system and associated behaviours [112]. E2 levels and dopaminergic activity also fluctuate with infradian rhythms, particularly in rodent models. Almey et al. provide evidence that E2 modulates DA receptor activity, which varies across the estrus cycle in rodents [113].

Furthermore, promising results from our laboratory suggest E2‐dependent alterations in circadian and infradian rhythm during the acquisition phase of NIC consumption. Specifically, intact females anticipate spontaneous awakening (OVX do not exhibit this behaviour) 2 h before NIC delivery, showing higher seeking behaviour during proestrus (unpublished data).

These findings underscore the intricate interplay between E2, circadian rhythms and dopaminergic activity, highlighting the importance of temporal regulation in shaping addiction‐related behaviours and vulnerabilities.

### 4.6 Implications and Future Directions

The reviewed studies highlight the critical role of ovarian hormones in addiction, particularly in female rodents, though several methodological gaps remain. Key limitations include inconsistent experimental designs and insufficient transparency in randomization, blinding and baseline characteristics.

These findings underscore the need for targeted prevention and treatment strategies, especially for females, who show distinct vulnerabilities to substance use disorders. Hormonal influences significantly affect drug‐seeking behaviour, reinforcement efficacy and relapse vulnerability, but many studies focus on a single phase of addiction, limiting a comprehensive understanding. Future research should address multiple phases to offer a more complete view of addiction's progression.

Molecular mechanisms underlying addiction are also underexplored, with only 54% of studies investigating them (Table 3). Expanding research in this area could contribute to developing pharmacological treatments. Additionally, younger females appear more vulnerable to addiction, highlighting the need for prevention strategies focused on adolescents.

Finally, circadian and infradian rhythms' impact on hormonal fluctuations should be considered, as these biological rhythms can influence drug‐seeking behaviour and stress responses, altering addiction vulnerability across the menstrual cycle and life stages.

## Author Contributions

L.V.‐M. conducted the literature search, analysed the data and elaborated tables and figures. G.A. and C.J.‐P. conducted the research, analysed the data, reviewed tables and figures and wrote the paper. C.J.‐P. also designed the study and elucidated the molecular mechanisms involved. T.M.‐J. edited the manuscript and provided feedback on the report. R.C.Z., Ó.L.‐F. and M.F.‐M. also provided feedback on the report. All authors read and approved the final manuscript.

## Conflicts of Interest

The authors declare no conflicts of interest.

## Supporting information


**Table S1.** Table summarizing the life stages, age ranges and species of animals used in the studies reviewed.

## Data Availability

The data that support the findings of this study are available from the corresponding author upon reasonable request.
