# Supplementary material for: Ovarian Hormones and Addictive Behaviour Vulnerability: Insights From Preclinical Studies
Source: Addict Biol. 2025 Jun 8;30(6):e70046. doi: 10.1111/adb.70046 (PMC12145796; doi:10.1111/adb.70046)
Supplement: Supplementary file 1 — Table S1. Table summarizing the life stages, age ranges and species of animals used in the studies reviewed. [file ADB-30-e70046-s001.docx]

**Supplemental table 1.** Table summarizing the life stages, age ranges, and species of animals used in the studies reviewed.

| **Reference** | **Life stage** | **Days old** | **Weight** | **Species** |
| --- | --- | --- | --- | --- |
| Ashirova *et al.,* (2020) | Adolescents | 9 weeks (63d) |  | Sprague Dawley rats |
| Henricks *et al.,* (2019) | Adolescents | 60 d |  | Sprague Dawley rats |
| Holly *et al.,* (2012) | Adolescents | 8 weeks (56d) |  | Long Evans rats |
| Jaster *et al.,* (2022) | Emerging Adulthood (young adult) | 8 (56d) -14 weeks |  | C57BL/6J mice |
| Ohia-Nwoko *et al.,* (2017) | Emerging adulthood | 16 (112d) - 12 weeks |  | BALB/c mice |
| Lazenka *et al.,* (2017) | Emerging adulthood |  | M: 300-350g  F: 250-300g | Sprague Dawley rats |
| Wiley *et al.,* (2011) | Peripubertal, adolescents | 27-38d; (65d) |  | Long Evans rats |
| D’Ottavio *et al.,* (2022) | Peripubertal | 5-6 weeks (42d) |  | Sprague Dawley rats |
| Kuhn *et al.,* (2022) | Juvenile | 5 weeks |  | (N/NIH-HS) rats |
| Calipari *et al.,* (2017) | Adolescents | 6-8weeks (56d) |  | C57BL/6J mice |
| Piza-Palma *et al.,* (2014) | Peripubertal | 21d+ 2w (35d) |  | C57BL/6J mice |
| Li *et al.,* (2012) | Juvenile | 26-28d |  | Long Evans rats |
| Algallal *et al., (*2019) | Emerging adulthood |  | M:225-250  F: 150-175 | Wistar rats |
| Cullity *et al.,* (2021) | Adolescents | 49d, 70d |  | n/s mice |
| Kerstetter *et al.,* (2011) | Adolescents | 9 weeks (63d) |  | Sprague Dawley rats |
| Rubino *et al.,* (2008) | Peripubertal | 28d start: 35-45d |  | Sprague Dawley rats |
| Ryan *et al.,* (2018) | Adolescents | 9 weeks (63) |  | Sprague Dawley rats |
| Wronikowska *et al.,* (2021) | Adolescents | 8 weeks (56) |  | Wistar rats |
| Xie *et al.,* (2019) | Adolescents | 9 weeks (63d) |  | C57BL/6J mice |
| Lacy *et al.,* (2016) | Emerging  adulthood | 70d + 1 week |  | N/S rats |
| Daiwile *et al.,* 2019 | Late adulthood |  | M: 450-600g  F: 350-500g | Long Evans rats |
| Reichel *et al.,* (2012) | Adolescents | 9 weeks (63d) |  | Long Evans rats |
| Towers *et al.,* (2021) | Emerging Adulthood |  | M: 370g  F: 270g | Sprague Dawley rats |
| Sutton *et al.,* (2021) | Peripubertal | 5-7weeks (40d) |  | Long Evans rats |
| Mohammadian & Miladi‐Gorji, (2019) | Emerging adulthood; middle adulthood | Group 1: 16-20 weeks  Group 2: 72-76 weeks |  | Wistar rats |
| Bossert *et al.,* (2021) | Emerging adulthood |  | M: 250-350g  F: 175-225g | Sprague Dawley rats |
| Castelli *et al., (*2014) | Adolescents | 8 weeks |  | Lister Hooded rats |
| Maher *et al.,* (2022) | Adolescents | 8 weeks |  | Long evans rats |
| Smith *et al.,* (2021) | Peripubertal | 49d |  | Long Evans rats |
| Peterson *et al.,* (2016) | Emerging  adulthood | 12 weeks |  | Sprague Dawley rats |
| Yararbas y Pogun (2011) | - | Sin dato de peso/edad pero dice adultos |  | Sprague Dawley rats |
| Winsauer *et al.,* (2010) | Emerging  adulthood | 30-75d |  | Long Evans rats |
| Fattore *et al.,* (2010) | Adolescents | 8 (56d)-9 weeks |  | Lister Hooded rats |
| Kerstetter *et al.,* (2012) | Adolescents | 8 weeks |  | Sprague Dawley rats |
| Torres *et al.,* (2013) | Peripubertal, Adolescents | 60-75d y 28-45d |  | Wistar rats |
| Zovkic y McCormick (2019) | Adolescents | 60d |  | Long Evans rats |
| Towers *et al.,* (2023) | “Sexually mature”-Equals to Emerging adulthood | N/S | N/S | Sprague Dawley rats |
| Cummings *et al.,* (2014) | Adolescents | 50-55d |  | Sprague Dawley rats |
| Sedki *et al.,* (2015) | Adolescents | 8-9 weeks |  | Long Evans rats |
| Lacy *et al.,* (2019) | Emerging adulthood | 10 weeks (75d) |  | Long Evans rats |
| Satta *et al.,* (2018) | Adolescents | 8 weeks |  | C57BL/6J mice |
| Bertholomey *et al.,* (2016) | Emerging  adulthood | 30d-120 inicia AA |  | Sprague Dawley rats |
| Feltenstein *et al.,* (2011) | Adolescents | 59-67d, 57-74d |  | Sprague Dawley rats |
| Swalve *et al.,* (2016) | Emerging  adulthood | 9 (63 d)-10 weeks |  | Wistar rats |
| Phillips *et al.,* (2019) | Emerging adulthood | 10-12 weeks |  | C57BL6 mice |
| Anker y Carroll (2010) | Emerging adulthood | 10 weeks |  | Wistar rats |

*Neonatal 0-7 d, Infantile 7-21 d, Juvenile/childhood 21-35 d, Peripubertal 35-55 d, Adolescents 55-70 d, Emerging adulthood 70-150 d, Young adulthood 150-300 d, Middle adulthood 300-600 d, Older adulthood 600-730 d, late adulthood 730 + d*
